# Roles and Regulations of Acid Invertases in Plants: Current Knowledge and Future Perspectives

**DOI:** 10.3390/plants14030320

**Published:** 2025-01-22

**Authors:** Jia Liu, Yuan Cheng, Meiying Ruan, Qingjing Ye, Rongqing Wang, Zhuping Yao, Guozhi Zhou, Zhimiao Li, Chenxu Liu, Hongjian Wan

**Affiliations:** 1 State Key Laboratory for Quality and Safety of Agro-Products, Institute of Vegetables, China-Australia Research Centre for Crop Improvement, Zhejiang Academy of Agricultural Sciences, Hangzhou 310021, China; liu-l-jia@163.com (J.L.); chengyuan@zaas.ac.cn (Y.C.); ruanmy@zaas.ac.cn (M.R.); yeqj@zaas.ac.cn (Q.Y.); wangrq@zaas.ac.cn (R.W.); yaozp@zaas.ac.cn (Z.Y.); zhougz@zaas.ac.cn (G.Z.); zhimiaoli@zaas.ac.cn (Z.L.); liuchenxu@zaas.ac.cn (C.L.); 2 Wulanchabu Academy of Agricultural and Forestry Sciences, Wulanchabu 012000, China; 3 China-Australia Research Centre for Crop Improvement, Zhejiang Academy of Agricultural Sciences, Hangzhou 310021, China

**Keywords:** acid invertases (Ac-Invs), plant physiology, hormonal regulation, environmental stress responses

## Abstract

Acid invertases (Ac-Invs) are crucial enzymes in plant physiology, regulating sucrose metabolism and hydrolyzing sucrose into glucose and fructose. These sugars serve not only as energy sources and structural components but also as signaling molecules, influencing diverse developmental processes, including seed and fruit growth, flowering, and stress responses. Ac-Invs are classified into cell wall invertases (CWINs) and vacuolar invertases (VINs) based on their subcellular localization, with both playing distinct roles in sucrose unloading, osmotic regulation, and sugar accumulation. Recent studies have also highlighted their involvement in abiotic stress adaptation and hormonal regulation, emphasizing their central role in plant resilience and productivity. However, gaps remain in understanding their regulatory mechanisms, particularly their interactions with plant hormones, defective invertases, and responses to environmental stresses. This review summarizes the biochemical characteristics, functions, and regulatory mechanisms of Ac-Invs, providing insights into their evolutionary significance and potential applications in crop improvement. Future research directions are proposed to elucidate unresolved questions and leverage Ac-Invs for enhancing agricultural sustainability.

## 1. Introduction

**Figure 1:**
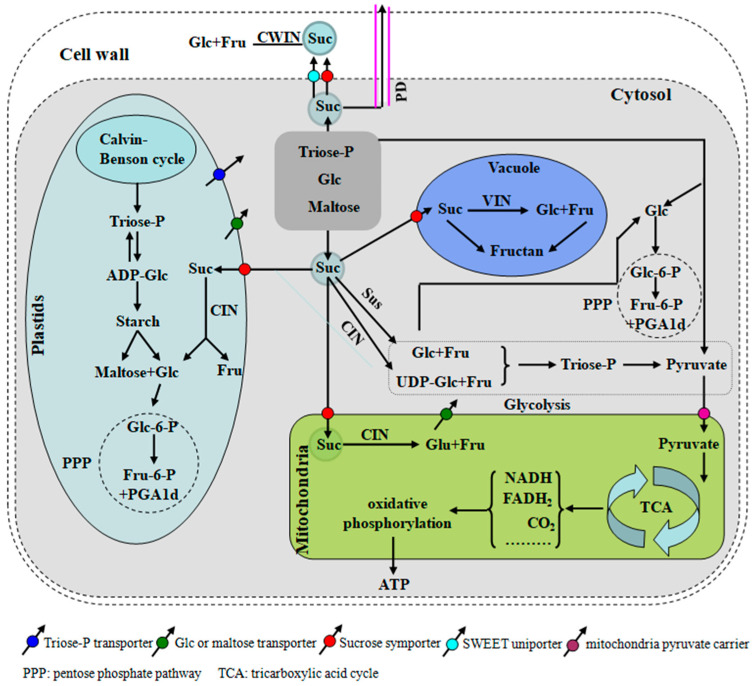
Sucrose unloading, transport, and metabolism in sink tissues. Suc is unloaded from the phloem into sink cells either apoplasmically or symplasmically. For the former, Suc could be hydrolyzed by cell wall invertase (CWIN) into glucose (Glc) and fructose (Fru). Cytosolic Suc may be taken up into vacuoles for hydrolysis by vacuolar invertase (VIN) and also hydrolyses into Glc and Fru by cytoplasmic invertase (CIN) or SUS used for glycolysis. The various transporters involved are also shown.

Sucrose (Suc) is the primary end product of photosynthesis in photoautotrophic bacteria and most higher plants. It is transported through the phloem from “source organs” (primarily photosynthetic tissues such as leaves) to “sink tissues” (non-photosynthetic tissues like roots, flowers, fruits, and seeds) [1,2,3]. In plant sink tissues, sucrose and the hexoses (glucose and fructose) derived from its hydrolysis provide both the carbon skeleton and energy necessary for fruit and seed development (Figure 1). They also act as signaling molecules, influencing growth and development by regulating the expression of various genes [4]. After being transported via the phloem, sucrose can be broken down into hexoses or their derivatives by two enzymes: sucrose synthase (SUS; EC 2.4.1.13) and invertase (INV; EC 3.2.1.26). SUS catalyzes the reversible conversion of sucrose into uridine diphosphate glucose (UDP-Glc) and fructose (Fru), whereas INV irreversibly hydrolyzes sucrose into glucose (Glc) and fructose (Fru) [5,6] (Figure 1).

Research has shown that SUS plays a crucial role in the development of sink tissues in crop species [7,8,9], primarily contributing to the biosynthesis of lipids, proteins, and carbohydrates [such as cellulose and starch] [10]. INV is essential for plant reproductive development [11,12] and is involved at various stages of plant growth, playing a critical role in defense responses to both abiotic and biotic stresses [13]. The importance of INV in plant growth and development, and its practical applications has sparked extensive research into its functions.

INV is found across microorganisms, plants, and animals. Based on their optimal pH values, INVs are divided into two major categories: Ac-Invs, with an optimal pH of 4.5 to 5.5, and alkaline/neutral invertases (N/A-Invs), with an optimal pH of 7.0 to 7.8 [14]. Based on subcellular localization, INVs can be classified into three types: cell wall invertase (CWIN), vacuolar invertase (VIN), and CIN (Figure 1). Both CWIN and VIN belong to the acid invertase and can hydrolyze sucrose and other β-fructan oligosaccharides, thus also being referred to as β-fructofuranosidases. CIN, however, is classified as an N/A-Invs [15,16]. CWIN and VIN share similar enzymatic properties, including comparable amino acid sequences and protein structures, making them closely related in clustering [17,18]. These enzymes can hydrolyze sucrose and other sugars containing β-fructofuranosidic linkages, such as raffinose and stachyose [19,20], and are susceptible to inhibition by heavy metal ions (Cu^2+^, Hg^2+^, Co^2+^, etc.).

In contrast, N/A-Invs do not hydrolyze β-fructan oligosaccharides but specifically catalyze the hydrolysis of sucrose. Their activity is not inhibited by heavy metal ions [21,22]. Ac-Invs are of particular interest due to their critical roles in plant growth, development, and adaptation to environmental challenges. This review will focus on the biological characteristics of plant Ac-Invs, summarizing their physicochemical properties, gene families, functions, and the regulation of their expression. The aim is to lay a theoretical foundation for further exploration of the functional characteristics of Ac-Invs.

## 2. Physicochemical Characteristics of Ac-Invs

In addition to hydrolyzing sucrose, both CWIN and VIN can also degrade other β-fructan oligosaccharides, earning them the designation of β-fructofuranosidases [18,19]. These enzymes play distinct and essential roles in plant physiology. Ac-Invs contain two conserved domains: an N-terminal domain (PF00251) and a C-terminal domain (PF08244). The N-terminal domain undergoes glycosylation and is classified under the “glycoside hydrolase family 32” (GH32). The protein structure consists of five β-spirals arranged in combination with a β-sandwich model, with the catalytic site located within the β-spirals. Three conserved motifs—DNPNG (Asp-Asn-Pro-Asn-Cys), FRDP (Phe-Arg-Asp-Pro), and WECPD (Trp-Glu-Cys-Pro-Asp)—are common to all β-fructofuranosidases and are crucial for the catalytic activity of Ac-Invs [16,23].

Ac-Invs exhibit a high affinity for their substrate, sucrose, with Km values ranging from 2 to 6 mM. VINs, in particular, show an even greater affinity for sucrose [19]. Studies on VINs in Japanese pears have demonstrated Km values of 4.58 and 3.33 mM for sucrose hydrolysis, with only 17% and 22% of this activity when hydrolyzing raffinose and stachyose, respectively [24]. However, some exceptions have been noted, such as apple VIN, which displays Km values of 2.9 mM for sucrose and 562 mM for stachyose, but lacks the ability to hydrolyze raffinose [25]. These findings suggest that VINs may have specific substrate preferences, highlighting their functional diversity across different plant species.

## 3. Identification and Expression of Acid Invertases in Plants

### 3.1. Acid Invertase Gene Families in Plant Species

Ac-Invs are generally believed to have originated in eukaryotes and aerobic bacteria [17]. In 1980, Berthelot isolated a substance from sugarcane that could decompose sucrose, producing a 1:1 mixture of dextro-fructose and levulose (glucose). This mixture was termed “invert sugar,” leading to the naming of the enzyme as “invertase” [21]. The first plant invertase was isolated from potatoes by Kastle et al. in 1903 [22]. A significant milestone came in 1990, when Sturm et al. successfully cloned the first plant acid invertase—the carrot cell wall invertase gene—marking the first cloning of Ac-Invs in higher plants [18]. Since then, invertases have been cloned from a variety of plants and tissues, including Arabidopsis [26], tomato [27], carrot [28], and maize [29]. These genes typically consist of 6 to 8 exons [30].

**Figure 2:**
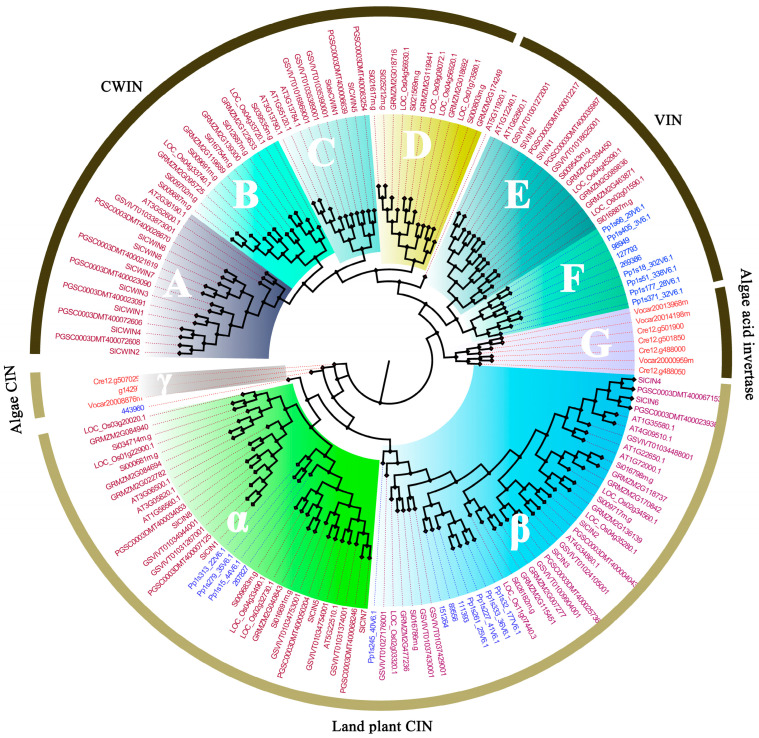
Molecular phylogenetic analysis of invertase (acid invertase and neutral/alkaline invertase) in different plant species used the Maximum Likelihood method. The evolutionary history was inferred by using the Maximum Likelihood method based on the Le_Gascuel_2008 model. The percentage of trees in which the associated taxa clustered together is shown next to the branches. Initial tree(s) for the heuristic search were obtained automatically by applying Neighbor-Join and BioNJ algorithms to a matrix of pairwise distances estimated using a JTT model, and then selecting the topology with superior log likelihood value. The tree is drawn to scale, with branch lengths measured in the number of substitutions per site. The analysis involved 88 amino acid sequences. All these members were divided into neutral/alkaline invertases and acid invertases, the former was subdivided into three groups (γ, α, and β), the latter was composed of seven groups (A, B, C, D, E, F and G).

With the rapid advancement of plant genome sequencing technologies, bioinformatics approaches are increasingly being used to identify and analyze Ac-Inv gene families at the whole-genome level. For instance, three genes encoding CWIN and three genes encoding VIN have been identified in tea [31], four CWIN and two VIN genes in *Arabidopsis* [32], nine CWIN and two VIN genes in rice [33], two CWIN and two VIN genes in *Dendrobium huoshanense* [34], two CWIN and two VIN genes in *Dendrobium officinale* [35], and five CWIN and three VIN genes in poplar [36]. The number of genes encoding Ac-Invs varies across species, with CWIN genes typically having more members than VIN genes. CWIN genes are absent in algae, while VIN genes are consistently present, albeit with varying numbers of gene members. Interestingly, VIN genes show higher conservation compared to CWIN genes. To further investigate the evolutionary relationships of plant invertases, we performed a phylogenetic analysis of invertase genes from various plant species. The analysis revealed a clear separation between acid and neutral/alkaline invertases, dividing them into two distinct groups. The acid invertases form a clade with algal acid invertases as the basal group, and the acid invertases in higher plants are further classified into two main subtypes: cell wall invertases (CWINs) and vacuolar invertases (VINs). The CWIN group is further divided into four subgroups (A-D), while the VIN group is divided into two subgroups (E,F) (Figure 2).

### 3.2. Expression and Regulation of Acid Invertase Genes in Plants

#### 3.2.1. Temporal–Spatial and Specific Expression

Sturm et al. [18] cloned the first gene encoding CWIN from carrot and found that its expression was almost identical across leaves, petioles, and roots during the primary root stage, suggesting that the CWIN gene in carrot exhibits developmental stage-specific expression. In contrast, two VIN genes were predominantly expressed in root tips and primary roots, indicating organ-specific expression [18]. Studies on the expression of the CWIN gene in tomatoes [37] revealed organ-specific patterns, with *Lin5* being specifically expressed in stamens and ovaries, and *Lin7* in pistils and pollen, suggesting that these two genes may play a role in flower development. On the other hand, *Lin6* and *Lin8* were primarily expressed in vegetative organs such as roots and leaves. Research on Arabidopsis also demonstrated temporal–spatial-specific expression of CWIN genes, with some genes being specifically expressed in leaf and shoot apical meristems, influencing flowering time and inflorescence number [38]. Similar patterns have been observed in other plants, such as the specific expression of a CWIN gene in the flower buds of lilies [39] and in flowers during the flowering period of carrots [40].

Investigation into the expression pattern of CWIN genes in cotton [41] showed that *GhCWIN1* was significantly expressed in the endosperm during the early stages of seed development, potentially playing a crucial role in early endosperm development. *VfCWIN* exhibited high expression in the testa of larger seeds in broad beans [42], and the deletion of a CWIN gene in maize resulted in smaller seed kernels [43], further emphasizing the importance of Ac-Invs in seed development. Since the testa is also a site for sucrose unloading, these findings suggest that Ac-Invs play a role in this pathway.

These studies indicate that the expression of Ac-Inv genes varies across different plants and developmental stages. The expression of the Ac-Invs family may be organ-specific or developmentally regulated, likely fulfilling distinct functions at different stages of plant growth and development.

#### 3.2.2. Regulation by Plant Hormones

In plants, not only sugars but also plant hormones play crucial roles in growth, development, and tissue differentiation. Studies have shown that hormones such as auxins, cytokinins (CTKs), gibberellins (GAs), and abscisic acid (ABA) can enhance the activity of Ac-Invs. For instance, after treating 30-day-old soybean seeds with appropriate concentrations of GA, Ac-Inv activity was found to increase in the seed coat but decrease in the cotyledons, suggesting that GA may promote sugar unloading in the seed coat and its subsequent allocation to the cotyledons. In tomatoes, GA induction resulted in the downregulation of *Lin7*, with its expression becoming pollen-specific [44]. Additionally, studies have demonstrated that ABA has a significant positive effect on Ac-Inv activity during various stages of grape fruit development, as well as during the mid-to-late stages of apple fruit development. However, the modes of action of ABA differ between the two fruits. Researchers treated 10-day-old corn seedlings with ABA and observed enhanced Ac-Inv activity in both roots and leaves [45]. The studies cited in this section have demonstrated that the expression of Ac-Inv genes in plants can be induced by exogenous hormone treatments, with GA and ABA typically leading to an increase in activity, though exceptions exist, such as in potatoes. Changes in Ac-Inv activity can also influence endogenous plant hormones. For example, the inhibition of tobacco CWIN activity has been shown to suppress the cytokinin-regulated mechanism that delays leaf senescence [46].

#### 3.2.3. Regulation by Environmental Stress

Plants often encounter various environmental stresses during growth, such as heavy rain, high temperatures, and snowfall. In response to these stresses, plants initiate specific responses to maintain cellular function, with invertases playing a key role in sugar metabolism. As crucial enzymes, Inv levels and activity are often induced or altered to support plant survival under stress conditions.

Inv’s response to environmental stresses varies across different plant tissues. Under drought conditions, for example, the activity of CWIN in mature corn leaves remains relatively unchanged, while the expression of a VIN gene (*Inr2*) is upregulated, leading to increased VIN activity and hexose accumulation. This helps to raise osmotic pressure and enhance water uptake [45]. In contrast, under water stress in corn flowers, both CWIN and VIN activities decrease, which impairs ovary development [47]. In cucumbers, *CsVI2* enhances drought tolerance in cucumber seedlings by regulating sucrose metabolism and increasing vacuolar invertase activity [48]. In tobacco, the researchers identified 36 NtINV genes, highlighting their roles in leaf development and stress tolerance, with NtNINV10 involved in drought and salinity responses [49].

Temperature fluctuations are another common environmental challenge that affect Ac-Inv activity. In potato tubers stored at low temperatures, sugar accumulation increases, and Ac-Inv activity correlates positively with sugar content [50]. A similar phenomenon is observed in sweet potatoes, where low temperatures also induce higher Ac-Inv activity [51]. However, heat stress reduces CWIN activity in tomato anther tissues, which may be linked to pollen sterility [52]. Li et al. further suggested that the tomato *Lin7* gene may play an important role in regulating the heat stress response [53], indicating that plant responses to temperature stresses vary based on the type of stress. In addition, recent studies highlight the critical role of vacuolar invertase genes in plant cold tolerance, uncovering regulatory mechanisms, gene family evolution, and alternative pathways for adaptation to low temperatures [54,55,56].

Ac-Invs are also involved in the plant’s response to mechanical damage. For example, when sweet potato tubers are sliced, there is a significant increase in VIN activity [57]. In carrots, damage to the main root results in a marked increase in CWIN expression and activity at the injury site [18]. Additionally, salt stress can affect Ac-Inv activity; in white lupin, VIN activity increases under moderate NaCl stress but decreases under higher NaCl concentrations [58].

## 4. Functions of Acid Invertase Genes in Plants

CWIN and VIN are capable of hydrolyzing sucrose into glucose and fructose. These products—sucrose and the hexoses (glucose and fructose) generated from its breakdown—serve not only as critical energy and nutrient sources for plant growth and development but also function as signaling molecules that regulate gene expression, influencing processes such as fruit and seed development [37]. A growing body of evidence indicates that Ac-Invs play vital roles in plant growth, yield, and quality, as well as in sugar accumulation and the plant’s responses to both biotic and abiotic stresses [59].

### 4.1. Participation in Sucrose Unloading and Transport Regulation

Sucrose is the primary end product of photosynthesis in higher plants and serves as the major form of long-distance transport. After being transported from source organs to sink organs, sucrose is unloaded into fruits. This unloading process occurs via two main pathways: apoplastic unloading and symplastic unloading. In apoplastic unloading, sucrose can enter sink organ cells through proton pumps (H^+^-ATPase) or transporters on the plasma membrane. Alternatively, sucrose can be hydrolyzed by CWIN into hexoses, which are then transported into the cell via hexose carriers. Symplastic unloading involves the movement of sucrose through plasmodesmata into cells, where it is typically catalyzed by SUS or CIN. If sucrose enters the vacuole, the process is catalyzed by VIN [4].

CWIN is an insoluble protein that irreversibly hydrolyzes sucrose when it is unloaded through the apoplastic pathway. This enzymatic activity reduces the sucrose concentration in the apoplast, thereby maintaining the concentration gradient necessary for efficient unloading. The hexoses produced by CWIN are then transported into the cell via hexose transporters [4]. Studies have shown that CWIN genes are highly expressed in regions of phloem unloading, such as the seed coat of broad beans [42], and the pericarp during walnut fruit development [60]. These findings suggest that CWIN plays a significant role in sucrose unloading, with pericarp development potentially being linked to phloem unloading.

The efficiency of sucrose transport is influenced by the strength of sink organs, which determines their attraction for sucrose. The activity of the key enzymes involved in carbohydrate metabolism is a critical biochemical indicator of sink strength. For example, in grapes, VIN activity is low during flowering but increases as berry (sink organ) development begins, reaching a peak two weeks later before declining to near zero [38]. Studies on the maize mutant min1, which produces smaller grains, suggest that the absence of a gene encoding CWIN impairs sucrose transport to the grain (sink organ) [43].

### 4.2. Participation in Plant Growth and Development

Sucrose and its metabolites provide essential energy and nutrients for plant life processes, while sugars also play a role in regulating cell differentiation and growth [40]. Studies have shown that invertase exhibits high enzymatic activity in the rapidly growing or meristematic tissues of plants, including floral buds, root tips, young leaves, and fruits [61,62]. CINs function as “maintenance enzymes” and are critical for root and reproductive development. When CIN activity was reduced in *Arabidopsis thaliana* mutants [63] and in rice [64], root growth was inhibited, and flowering abnormalities occurred in rice.

Numerous studies have demonstrated that CWIN regulates the ratio of sucrose to hexoses in the endosperm and embryo, thereby controlling the carbohydrate composition and seed growth [13]. In maize, a natural mutant with a deletion of the *ZmCWIN2* gene affected endosperm cell mitosis, leading to visible wrinkling in seeds. The specific expression of *ZmCWIN2* in the endosperm suggests that hexoses produced by CWIN not only serve as nutrients but also function as signals that regulate cell division and differentiation [38]. By modulating the expression of the CWIN gene in rice, seed size could be altered accordingly [65]. New research has shown that the vacuolar invertase *OsVIN2*, encoded by rice, alters sugar metabolism, thereby affecting grain size [66], indicating that Ac-Invs play important regulatory roles in rice grain development. Additionally, in broad beans, the *VfCWIN1* gene was expressed at higher levels in the seed coat of larger seeds [42]. Comparisons of two cowpea pod varieties with different degrees of fullness revealed a correlation between seed and pod wall fullness and CWIN activity [67]. These results suggest that CWIN activity and expression levels are important for seed growth and development.

Furthermore, studies have demonstrated that CWIN influences pollen development. The suppression of the CWIN gene (*Nin88*) specifically expressed in tobacco anthers led to impaired pollen development and male sterility [68]. The cold-induced suppression of the anther-specific CWIN gene (*OSINV4*) in rice reduced CWIN activity, impaired sucrose accumulation, and caused pollen sterility [69]. Under heat stress in tomatoes, CWIN metabolism was disrupted, leading to decreased pollen viability and a reduced number of released pollen grains [70]. These findings suggest that under various abiotic stress conditions, reduced CWIN enzyme activity decreases hexose production, hindering pollen development and contributing to male sterility or low pollen viability, highlighting the important role of CWIN in pollen development.

Additionally, CWIN has been found to delay leaf cell senescence. During leaf senescence, nutrient and sugar mobilization occur, and cytokinins play a key role in this process. Lara et al. [46] demonstrated that cytokinins delay senescence through CWIN, providing insight into the potential molecular mechanisms that delay leaf senescence. When CWIN activity increased, it was found to be effective in delaying leaf senescence.

### 4.3. Participation in Osmotic Regulation

Plant growth is accompanied by the accumulation of hexoses, which serve as both carbon sources and nutrients, while also providing the driving force for cell elongation. Ac-Inv plays a key role in maintaining osmotic pressure, enhancing cell wall pliability, and promoting plant growth and cell elongation. VIN catalyzes the hydrolysis of sucrose into two molecules of hexose, which increases the osmotic pressure within the cell and facilitates cell elongation. When VIN is mutated in Arabidopsis, the elongation zone cells in the roots are significantly shortened [71]. In oat coleoptiles, VIN activity is approximately three times higher than in the mesocotyls, and in sunflower hypocotyls, VIN activity is closely associated with elongation, likely due to its effect on osmotic pressure [72]. In cotton, VIN activity is positively correlated with the rapid elongation of fiber cells [41]. These findings highlight the crucial role of VIN in cell elongation, particularly through its involvement in osmotic regulation.

### 4.4. Influence on Sugar Accumulation

The sugars in fruits primarily accumulate in the vacuoles. For example, in orange pulp, most of the hexoses and all of the sucrose are stored in the vacuole [73], and the same is true for hexoses in apples [74]. VIN hydrolyzes sucrose into hexoses within the vacuole, playing a key role in the distribution and accumulation of sucrose and hexoses. When comparing two cucumber genotypes—sucrose-accumulating and hexose-accumulating—it was found that VIN activity was lower in the sucrose-accumulating fruit types during maturation, while VIN activity increased significantly in the hexose-accumulating types [75]. A similar phenomenon was observed in tomatoes: in wild tomatoes (sucrose-accumulating), VIN gene transcription levels were very low, and enzyme activity was minimal in developing fruits. In cultivated tomatoes (hexose-accumulating), VIN activity increased rapidly during fruit development, leading to a substantial accumulation of hexoses and a reduction in the sucrose content [76]. During grape berry development, VIN activity increased progressively, indicating a strong correlation between hexose accumulation and VIN activity in grapes [60].

This suggests that VIN plays a crucial role in promoting hexose accumulation and regulating the distribution of sucrose and hexoses during fruit development. Klann et al. used antisense gene technology to suppress VIN expression in cultivated tomatoes (*TIV1*), altering the concentrations of sucrose and hexoses in transgenic tomato fruits [77]. This finding supports the idea that sucrose accumulation is favored under conditions of low VIN activity or expression. In different litchi cultivars, the sucrose and hexose contents were closely linked to VIN activity, with cultivars exhibiting higher VIN activity showing a lower sucrose content [78]. Similarly, in potatoes, the suppression of VIN activity altered the soluble sugar components in tubers stored at low temperatures, decreasing the hexose content [79]. Collectively, these studies demonstrate that VIN regulates sugar accumulation, balancing the carbohydrate composition in fruits and tubers and influencing the distribution of sucrose and hexoses.

### 4.5. Defective or Nonfunctional Invertases

Wan et al. [80] demonstrated that defective or nonfunctional invertases are commonly found in higher plants. Their study revealed that nearly half of the Ac-Inv genes in four different plant species lack functionality due to the absence of the essential NDPN (Asn-Asp-Pro-Asn) sequence, which is crucial for sucrose hydrolysis. Additionally, some Ac-Inv genes exhibit substitutions at Asp239, a characteristic of defective invertases that are unable to hydrolyze sucrose. For example, the Nin88 gene, part of the CWIN family in tobacco, was found to carry mutations at Trp47 and Asp239, leading to the loss of its sucrose hydrolysis capability. This finding contradicts earlier assumptions that *Nin88* was a functional invertase gene [81]. Interestingly, in vitro experiments suggest that *Nin88* may still regulate sucrose hydrolysis by competitively binding with active invertases or their inhibitors, as well as interacting with the cell wall [81].

## 5. Concluding Remarks and Future Perspectives

Research on Ac-Invs has significantly enhanced our understanding of their crucial roles in plant physiology, including growth, development, osmoregulation, sugar accumulation, and responses to environmental stresses. However, several key questions remain unanswered, highlighting areas for future investigation. Specifically, the precise mechanisms by which plant hormones regulate Ac-Invs, the functions of defective invertases, and the pathways involved in stress responses remain incompletely understood. Addressing these gaps will require an integrated approach, combining genetic, biochemical, and genomic techniques. Future research should focus on the following areas to further our understanding of Ac-Invs:

– Elucidating Hormonal Signaling Pathways: Developing comprehensive models to explain how plant hormones interact with Ac-Invs to regulate plant growth, development, and responses to stress. This could involve transcriptomic and proteomic approaches to identify the hormone-responsive elements and post-translational modifications of Ac-Invs.
– Investigating the Role of Defective Invertases: Exploring the functions of defective invertases and their interactions with other cellular proteins. This could be achieved through the generation of knockout or knockdown mutants, followed by phenotype analysis to understand the biological implications of nonfunctional invertases.
– Characterizing Stress Response Mechanisms: Investigating the molecular mechanisms by which Ac-Invs mediate plant responses to environmental stresses. Research should focus on how Ac-Invs help maintain cellular homeostasis under stress conditions. This may involve studying the expression and activity of Ac-Invs in plants subjected to various stress factors such as drought, salinity, and temperature extremes.
– Comparative Genomics and Evolutionary Studies: Conducting comparative genomic analyses to explore the evolutionary relationships between Ac-Invs in different plant species. This could provide insights into the conservation and divergence of Ac-Invs and their roles in plant adaptation and speciation.
– Translational Research for Crop Improvement: Applying the findings from fundamental research to develop crops with improved yields, stress tolerance, and nutritional quality. This could involve genetic engineering strategies to modulate Ac-Invs expression or activity in economically important crops, thereby enhancing agricultural productivity and sustainability.

By addressing these research directions, we can gain a more comprehensive understanding of the diverse roles of Ac-Invs in plant biology and use this knowledge to improve agricultural practices, enhance crop resilience, and promote food security.
